# 2-Bromo-*N*-(4-bromo­phen­yl)acetamide

**DOI:** 10.1107/S1600536809028219

**Published:** 2009-07-22

**Authors:** B. Thimme Gowda, Ingrid Svoboda, Sabine Foro, P. A. Suchetan, Hartmut Fuess

**Affiliations:** a Department of Chemistry, Mangalore University, Mangalagangotri 574 199, Mangalore, India; b Institute of Materials Science, Darmstadt University of Technology, Petersenstrasse 23, D-64287 Darmstadt, Germany

## Abstract

In the title compound, C_8_H_7_Br_2_NO, the conformation of the N—H bond is *anti* to both the carbonyl and C—Br bonds in the side chain. In the crystal structure, mol­ecules are packed into supra­molecular chains along the *c* axis by N—H⋯O hydrogen bonds.

## Related literature

For the preparation of the title compound, see: Gowda *et al.* (2003 ▶). For related structures, see: Andreetti *et al.* (1968 ▶); Gowda *et al.* (2007**a* ▶,b* ▶,*c* ▶).
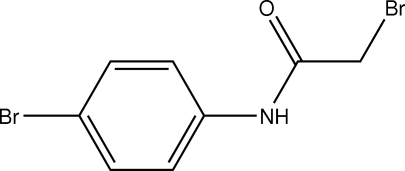


## Experimental

### 

#### Crystal data

C_8_H_7_Br_2_NO
*M* *_r_* = 292.97
Monoclinic, 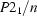

*a* = 4.4987 (3) Å
*b* = 23.152 (1) Å
*c* = 9.1098 (5) Å
β = 99.713 (6)°
*V* = 935.22 (9) Å^3^
*Z* = 4
Mo *K*α radiation
μ = 8.62 mm^−1^
*T* = 303 K
0.50 × 0.20 × 0.14 mm

#### Data collection

Oxford Diffraction Xcalibur diffractometer with a Sapphire CCD detector
Absorption correction: multi-scan (*CrysAlis RED*; Oxford Diffraction, 2009 ▶) *T*_min_ = 0.078, *T*_max_ = 0.299
3065 measured reflections
1661 independent reflections
1415 reflections with *I* > 2σ(*I*)
*R*_int_ = 0.023

#### Refinement

*R*[*F* ^2^ > 2σ(*F* ^2^)] = 0.049
*wR*(*F* ^2^) = 0.127
*S* = 0.99
1661 reflections
109 parameters
H-atom parameters constrained
Δρ_max_ = 0.73 e Å^−3^
Δρ_min_ = −0.68 e Å^−3^

### 

Data collection: *CrysAlis CCD* (Oxford Diffraction, 2009 ▶); cell refinement: *CrysAlis RED* (Oxford Diffraction, 2009 ▶); data reduction: *CrysAlis RED*; program(s) used to solve structure: *SHELXS97* (Sheldrick, 2008 ▶); program(s) used to refine structure: *SHELXL97* (Sheldrick, 2008 ▶); molecular graphics: *PLATON* (Spek, 2009 ▶); software used to prepare material for publication: *SHELXL97*.

## Supplementary Material

Crystal structure: contains datablocks I, global. DOI: 10.1107/S1600536809028219/tk2505sup1.cif

Structure factors: contains datablocks I. DOI: 10.1107/S1600536809028219/tk2505Isup2.hkl

Additional supplementary materials:  crystallographic information; 3D view; checkCIF report

## Figures and Tables

**Table 1:** Hydrogen-bond geometry (Å, °)

*D*—H⋯*A*	*D*—H	H⋯*A*	*D*⋯*A*	*D*—H⋯*A*
N1—H1*N*⋯O1^i^	0.86	2.11	2.925 (6)	157

Symmetry code: (i) 
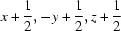
.

**Fig. 1.**
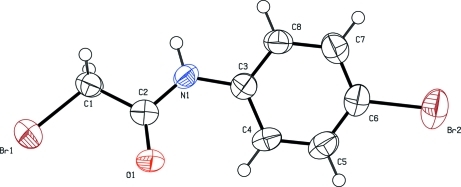
Molecular structure of (I), showing the atom labelling scheme. Displacement ellipsoids are drawn at the 50% probability level and H atoms are represented as small spheres of arbitrary radii.

**Fig. 2.**
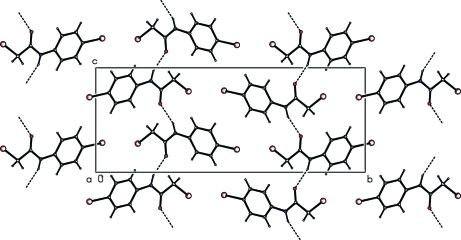
Molecular packing of (I) with hydrogen bonds shown as dashed lines.

## supplementary crystallographic information

### Comment

As part of a study of the effect of the ring and the side chain substituents on the structures of *N*-aromatic amides (Gowda *et al.*, 2007*a, b, c*), in the present work, the structure of 2-bromo-*N*-(4-bromophenyl)acetamide (I) has been determined (Fig. 1). The conformation of the N—H bond is *anti* to both the C=O and the C—Br bonds in the side chain, similar to that observed in 2-chloro-*N*-(4-chlorophenyl)acetamide (Gowda *et al.*, 2007*c*), *N*-(4-bromophenyl)acetamide (Andreetti *et al.*, 1968), and other amides (Gowda *et al.*, 2007*a, b*).

The crystal packing shows N1—H1N···O1 hydrogen bonds (Table 1) that lead to the formation of molecular chain along the *c*-axis (Fig. 2).

### Experimental

Compound (I) was prepared from 4-bromoaniline and bromoacetylchloride according to the literature method (Gowda *et al.*, 2003). Single crystals were obtained by slow evaporation of an ethanolic solution of (I) held at room temperature.

### Refinement

The H atoms were positioned with idealized geometry using a riding model [N—H = 0.86 Å, C—H = 0.93—0.97 Å], and were refined with isotropic displacement parameters (set to 1.2 times of the *U*_eq_ of the parent atom).

### Crystal data

**Table:**

C_8_H_7_Br_2_NO	*F*(000) = 560
*M**_r_* = 292.97	*D*_x_ = 2.081 Mg m^−^^3^
Monoclinic, *P*2_1_/*n*	Mo *K*α radiation, λ = 0.71073 Å
Hall symbol: -P 2yn	Cell parameters from 2250 reflections
*a* = 4.4987 (3) Å	θ = 2.9–27.8°
*b* = 23.152 (1) Å	µ = 8.62 mm^−^^1^
*c* = 9.1098 (5) Å	*T* = 303 K
β = 99.713 (6)°	Needle, colourless
*V* = 935.22 (9) Å^3^	0.50 × 0.20 × 0.14 mm
*Z* = 4	

### Data collection

**Table:**

Oxford Diffraction Xcalibur diffractometer with a Sapphire CCD detector	1661 independent reflections
Radiation source: fine-focus sealed tube	1415 reflections with *I* > 2σ(*I*)
graphite	*R*_int_ = 0.023
Rotation method data acquisition using ω and φ scans	θ_max_ = 25.4°, θ_min_ = 2.9°
Absorption correction: multi-scan (*CrysAlis RED*; Oxford Diffraction, 2009)	*h* = −5→4
*T*_min_ = 0.078, *T*_max_ = 0.299	*k* = −16→27
3065 measured reflections	*l* = −10→9

### Refinement

**Table:**

Refinement on *F*^2^	Primary atom site location: structure-invariant direct methods
Least-squares matrix: full	Secondary atom site location: difference Fourier map
*R*[*F*^2^ > 2σ(*F*^2^)] = 0.049	Hydrogen site location: inferred from neighbouring sites
*wR*(*F*^2^) = 0.127	H-atom parameters constrained
*S* = 0.99	*w* = 1/[σ^2^(*F*_o_^2^) + (0.0581*P*)^2^ + 4.1384*P*] where *P* = (*F*_o_^2^ + 2*F*_c_^2^)/3
1661 reflections	(Δ/σ)_max_ = 0.001
109 parameters	Δρ_max_ = 0.73 e Å^−^^3^
0 restraints	Δρ_min_ = −0.68 e Å^−^^3^

### Special details

**Table:**

Geometry. All e.s.d.'s (except the e.s.d. in the dihedral angle between two l.s. planes) are estimated using the full covariance matrix. The cell e.s.d.'s are taken into account individually in the estimation of e.s.d.'s in distances, angles and torsion angles; correlations between e.s.d.'s in cell parameters are only used when they are defined by crystal symmetry. An approximate (isotropic) treatment of cell e.s.d.'s is used for estimating e.s.d.'s involving l.s. planes.
Refinement. Refinement of *F*^2^ against ALL reflections. The weighted *R*-factor *wR* and goodness of fit *S* are based on *F*^2^, conventional *R*-factors *R* are based on *F*, with *F* set to zero for negative *F*^2^. The threshold expression of *F*^2^ > σ(*F*^2^) is used only for calculating *R*-factors(gt) *etc*. and is not relevant to the choice of reflections for refinement. *R*-factors based on *F*^2^ are statistically about twice as large as those based on *F*, and *R*- factors based on ALL data will be even larger.

### Fractional atomic coordinates and isotropic or equivalent isotropic displacement parameters (Å^2^)

**Table:**

	*x*	*y*	*z*	*U*_iso_*/*U*_eq_	
C1	0.7460 (14)	0.2109 (3)	0.3871 (6)	0.0489 (15)	
H1A	0.6432	0.2287	0.4610	0.059*	
H1B	0.9218	0.1909	0.4395	0.059*	
C2	0.8475 (13)	0.2577 (3)	0.2905 (6)	0.0400 (13)	
C3	1.1568 (13)	0.3474 (2)	0.3295 (6)	0.0388 (12)	
C4	1.0573 (14)	0.3743 (3)	0.1927 (6)	0.0445 (14)	
H4	0.9070	0.3573	0.1236	0.053*	
C5	1.1831 (16)	0.4263 (3)	0.1603 (7)	0.0535 (16)	
H5	1.1158	0.4444	0.0697	0.064*	
C6	1.4056 (14)	0.4510 (3)	0.2609 (7)	0.0473 (15)	
C7	1.5067 (15)	0.4251 (3)	0.3949 (7)	0.0538 (16)	
H7	1.6576	0.4424	0.4631	0.065*	
C8	1.3832 (14)	0.3731 (3)	0.4283 (7)	0.0476 (14)	
H8	1.4542	0.3553	0.5189	0.057*	
N1	1.0306 (11)	0.2954 (2)	0.3747 (5)	0.0397 (11)	
H1N	1.0770	0.2870	0.4677	0.048*	
O1	0.7725 (10)	0.26109 (19)	0.1567 (4)	0.0529 (11)	
Br1	0.48101 (16)	0.15590 (3)	0.27451 (8)	0.0576 (3)	
Br2	1.5669 (2)	0.52368 (3)	0.21830 (9)	0.0722 (3)	

### Atomic displacement parameters (Å^2^)

**Table:**

	*U*^11^	*U*^22^	*U*^33^	*U*^12^	*U*^13^	*U*^23^
C1	0.048 (3)	0.063 (4)	0.033 (3)	−0.015 (3)	0.000 (3)	−0.002 (3)
C2	0.038 (3)	0.044 (3)	0.038 (3)	0.007 (3)	0.007 (2)	−0.001 (3)
C3	0.044 (3)	0.037 (3)	0.037 (3)	0.004 (2)	0.012 (2)	−0.005 (2)
C4	0.053 (4)	0.050 (3)	0.029 (3)	0.004 (3)	0.004 (3)	0.003 (3)
C5	0.072 (4)	0.048 (4)	0.042 (3)	0.007 (3)	0.014 (3)	0.006 (3)
C6	0.058 (4)	0.038 (3)	0.053 (4)	−0.001 (3)	0.027 (3)	−0.003 (3)
C7	0.054 (4)	0.057 (4)	0.052 (4)	−0.012 (3)	0.012 (3)	−0.008 (3)
C8	0.048 (3)	0.052 (4)	0.041 (3)	−0.001 (3)	0.003 (3)	0.001 (3)
N1	0.051 (3)	0.039 (2)	0.028 (2)	−0.001 (2)	0.007 (2)	0.006 (2)
O1	0.072 (3)	0.054 (3)	0.030 (2)	−0.012 (2)	−0.001 (2)	−0.0005 (19)
Br1	0.0632 (5)	0.0541 (4)	0.0528 (4)	−0.0127 (3)	0.0024 (3)	−0.0073 (3)
Br2	0.1010 (7)	0.0456 (4)	0.0785 (6)	−0.0099 (4)	0.0392 (5)	−0.0006 (3)

### Geometric parameters (Å, °)

**Table:**

C1—C2	1.513 (8)	C4—H4	0.9300
C1—Br1	1.919 (6)	C5—C6	1.363 (9)
C1—H1A	0.9700	C5—H5	0.9300
C1—H1B	0.9700	C6—C7	1.367 (9)
C2—O1	1.211 (7)	C6—Br2	1.898 (6)
C2—N1	1.349 (7)	C7—C8	1.381 (9)
C3—C8	1.376 (8)	C7—H7	0.9300
C3—C4	1.398 (8)	C8—H8	0.9300
C3—N1	1.421 (7)	N1—H1N	0.8600
C4—C5	1.382 (9)		
			
C2—C1—Br1	112.6 (4)	C6—C5—C4	120.2 (6)
C2—C1—H1A	109.1	C6—C5—H5	119.9
Br1—C1—H1A	109.1	C4—C5—H5	119.9
C2—C1—H1B	109.1	C5—C6—C7	120.8 (6)
Br1—C1—H1B	109.1	C5—C6—Br2	119.8 (5)
H1A—C1—H1B	107.8	C7—C6—Br2	119.3 (5)
O1—C2—N1	124.7 (6)	C6—C7—C8	119.6 (6)
O1—C2—C1	124.9 (5)	C6—C7—H7	120.2
N1—C2—C1	110.4 (5)	C8—C7—H7	120.2
C8—C3—C4	118.8 (6)	C3—C8—C7	120.9 (6)
C8—C3—N1	117.7 (5)	C3—C8—H8	119.6
C4—C3—N1	123.5 (5)	C7—C8—H8	119.6
C5—C4—C3	119.8 (6)	C2—N1—C3	128.3 (5)
C5—C4—H4	120.1	C2—N1—H1N	115.8
C3—C4—H4	120.1	C3—N1—H1N	115.8
			
Br1—C1—C2—O1	0.2 (8)	Br2—C6—C7—C8	178.0 (5)
Br1—C1—C2—N1	179.5 (4)	C4—C3—C8—C7	1.3 (9)
C8—C3—C4—C5	−1.2 (9)	N1—C3—C8—C7	−176.9 (6)
N1—C3—C4—C5	177.0 (5)	C6—C7—C8—C3	−0.9 (10)
C3—C4—C5—C6	0.6 (9)	O1—C2—N1—C3	4.1 (9)
C4—C5—C6—C7	−0.3 (10)	C1—C2—N1—C3	−175.1 (5)
C4—C5—C6—Br2	−177.8 (5)	C8—C3—N1—C2	−167.5 (6)
C5—C6—C7—C8	0.4 (10)	C4—C3—N1—C2	14.3 (9)

### Hydrogen-bond geometry (Å, °)

**Table:**

*D*—H···*A*	*D*—H	H···*A*	*D*···*A*	*D*—H···*A*
N1—H1N···O1^i^	0.86	2.11	2.925 (6)	157

Symmetry codes: (i) *x*+1/2, −*y*+1/2, *z*+1/2.
